# Dimeric structure of the uracil:proton symporter UraA provides mechanistic insights into the SLC4/23/26 transporters

**DOI:** 10.1038/cr.2017.83

**Published:** 2017-06-16

**Authors:** Xinzhe Yu, Guanghui Yang, Chuangye Yan, Javier L Baylon, Jing Jiang, He Fan, Guifeng Lu, Kazuya Hasegawa, Hideo Okumura, Tingliang Wang, Emad Tajkhorshid, Shuo Li, Nieng Yan

**Affiliations:** 1 State Key Laboratory of Membrane Biology, School of Life Sciences and School of Medicine, Tsinghua University, Beijing 100084, China; 2 Beijing Advanced Innovation Center for Structural Biology, Tsinghua-Peking Joint Center for Life Sciences, School of Life Sciences and School of Medicine, Tsinghua University, Beijing 100084, China; 3 Center for Biophysics and Quantitative Biology, Beckman Institute for Advanced Science and Technology, Department of Biochemistry, University of Illinois at Urbana-Champaign, Urbana, IL 61801, USA; 4 Protein Crystal Analysis Division, Japan Synchrotron Radiation Research Institute, 1-1-1 Kouto, Sayo-cho, Sayo-gun, Hyogo 679-5198, Japan; 5 College of Life Sciences, Sichuan University, Chengdu, Sichuan 610064, China

**Keywords:** uracil/proton symporter, alternating access, conformational change

## Abstract

The *Escherichia coli* uracil:proton symporter UraA is a prototypical member of the nucleobase/ascorbate transporter (NAT) or nucleobase/cation symporter 2 (NCS2) family, which corresponds to the human solute carrier family SLC23. UraA consists of 14 transmembrane segments (TMs) that are organized into two distinct domains, the core domain and the gate domain, a structural fold that is also shared by the SLC4 and SLC26 transporters. Here we present the crystal structure of UraA bound to uracil in an occluded state at 2.5 Å resolution. Structural comparison with the previously reported inward-open UraA reveals pronounced relative motions between the core domain and the gate domain as well as intra-domain rearrangement of the gate domain. The occluded UraA forms a dimer in the structure wherein the gate domains are sandwiched by two core domains. *In vitro* and *in vivo* biochemical characterizations show that UraA is at equilibrium between dimer and monomer in all tested detergent micelles, while dimer formation is necessary for the transport activity. Structural comparison between the dimeric UraA and the recently reported inward-facing dimeric UapA provides important insight into the transport mechanism of SLC23 transporters.

## Introduction

The nucleobase-ascorbate transporter (NAT) family, also known as the nucleobase-cation symporter-2 (NCS2) family, mediates the uptake of nucleobases in bacteria, fungi, plants, and animals, and L-ascorbic acid (vitamin C) in mammals^1,2^. The mammalian NAT family members include SVCT1 and SVCT2 (the sodium-dependent vitamin C transporter, encoded by *SLC23A1* and *SLC23A2* genes)^3,4,5,6,7^. While SVCT1 is expressed in many cell types for vitamin C homeostasis, SVCT2 only functions in metabolically active cells and SVCT2-null mice died shortly after birth^8^. In plants, the NAT homolog LPE1 (leaf permease 1) was thought to be a high-affinity transporter for xanthine and uric acid^9^. A number of bacterial and fungal NAT proteins have been characterized, exemplified by the uric acid-xanthine transporter UapA in the filamentous fungi *Aspergillus nidulans* and the uracil:proton symporter UraA in *Escherichia coli*^10,11,12,13,14,15^.

The architecture of the NAT family (or SLC23 in human) was originally elucidated from the crystal structure of UraA, which was captured in an inward-open conformation with substrate bound^16^. UraA comprises fourteen transmembrane segments (TMs) that are organized into a pair of “7 + 7” inverted repeats. The intertwined repeats constitute two distinctive domains, namely the core domain and the gate domain. The substrate uracil is sandwiched by the two domains and coordinated predominantly by residues in the core domain. An unusual feature of UraA is the presence of short anti-parallel β-strands formed by the middle segments of TM3 and TM10, which provide the primary accommodation for uracil^16^.

Ensuing bioinformatic and computational studies suggested that the anion exchanger 1 (AE 1, also known as SLC4A1, or Band3) may share the same structural fold as UraA despite their poor sequence homology, establishing UraA a model for the structural and mechanistic investigation of a broader range of transporter families^7,17^. Indeed, the subsequently reported crystal structures of Band3 and a plant SLC4 member the borate efflux transporter Bor1 from *Arabidopsis thaliana* (AtBor1), as well as the bacterial homologue of SLC26 transporter from *Deinococcus geothermalis* (SLC26Dg) revealed the same folds as UraA^18,19,20^. The structural similarity of the SLC23, SLC4, and SLC26 proteins suggests their potential evolutionary relevance. Recently the structure of another NAT member UapA was determined in an inward-facing conformation in the presence of xanthine^21^. Despite that the first structure of UraA reveals a monomer in the crystal packing, Band 3, UapA and AtBor1 all have homodimeric organization in the crystal structure and oligomerization of UapA was shown to be critical for its cellular trafficking and membrane localization^18,20,21,22^.

Mechanistic understanding of a transporter requires structural resolution of multiple conformations during an alternating access cycle^23^. Hereby we present the 2.5 Å crystal structure of uracil-bound UraA homodimer in an occluded conformation. Comparison with the inward-facing structures of UraA and UapA, supplemented by structure-guided biochemical characterization, provides important insight into the transport mechanism of UraA and the SLC23 transporters in general.

## Results

### Structure of the occluded UraA in complex with uracil

**Figure 1:**
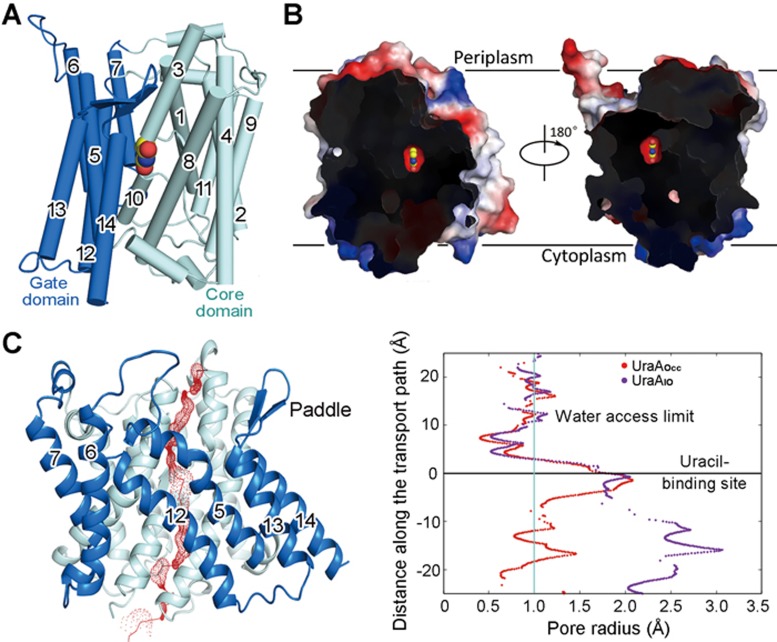
Structure of uracil-bound UraA in an occluded conformation. **(A)** The overall structure of a UraA protomer. Following the previous nomenclature, the 14 TMs are organized into a “core domain” (cyan) and a “gate domain” (blue). **(B)** The present structure of uracil-bound UraA represents an occluded state. Two opposite side views of the cut-open sections of UraA reveals that the bound uracil is insulated from either side of the membrane. The surface electrostatic potential was calculated by PyMol^44^. **(C)** The van der Waals interface between the core domain and the gate domain of UraA (red) was calculated with the program HOLE^24^. The radii along the potential transport path for the previous (purple) and the new (red) UraA structures are tabulated on the right. The previously reported inward-open and the new occluded structures are referred to as UraA_IO_ and UraA_Occ_, respectively.

After extensive screening for purification and crystallization conditions that may stabilize UraA in conformations other than the reported inward-open state (hereafter referred to as the UraA_IO_), we were able to crystallize the wild type (WT) UraA in the presence of 1.2% Fos-Choline 9 (FC-9) and 0.06% FC-11 (w/v) in the space group C222_1_ and collect X-ray diffraction data with resolutions beyond 2.4 Å at BL41XU, SPring-8. Molecular replacement and tungsten single-wavelength anomalous dispersion (W-SAD) were combined for phase determination. Compared to the previous UraA_IO_ structure in which residues 179-195 were built as poly-Ala due to poor resolution of this particular region, the new electron density map is of excellent quality and allows side group assignment of the consecutive polypeptide chain (residues 2-409). The final structure was refined to 2.5 Å resolution (Figure 1A; Supplementary information, Figure S1A and S1B, Table S1).

An evident difference of the two structures exists at the connecting segment between TMs 13 and 14, which forms a flexible loop in the previous UraA_IO_ structure, but a more rigid β-hairpin in the new structure (Figure 1A). After refinement of the protein structure, an omit electron density map corresponding to uracil was unambiguously recognized between the core domain (TMs 1-4 and 8-11) and the gate domain (TMs 5-7 and 12-14) (Supplementary information, Figure S1C). Solvent accessibility analysis using the program HOLE^24^ shows that the bound uracil is occluded from both sides of the membrane (Figure 1B and 1C). Therefore, the new structure of UraA represents a substrate-bound and occluded conformation. To simplify illustration, we will refer this state as UraA_Occ_. To simplify structural illustrations, we name the β hairpin between TMs 13 and 14 as “the paddle”.

### Conformational changes between UraA_IO_ and UraA_Occ_

**Figure 2:**
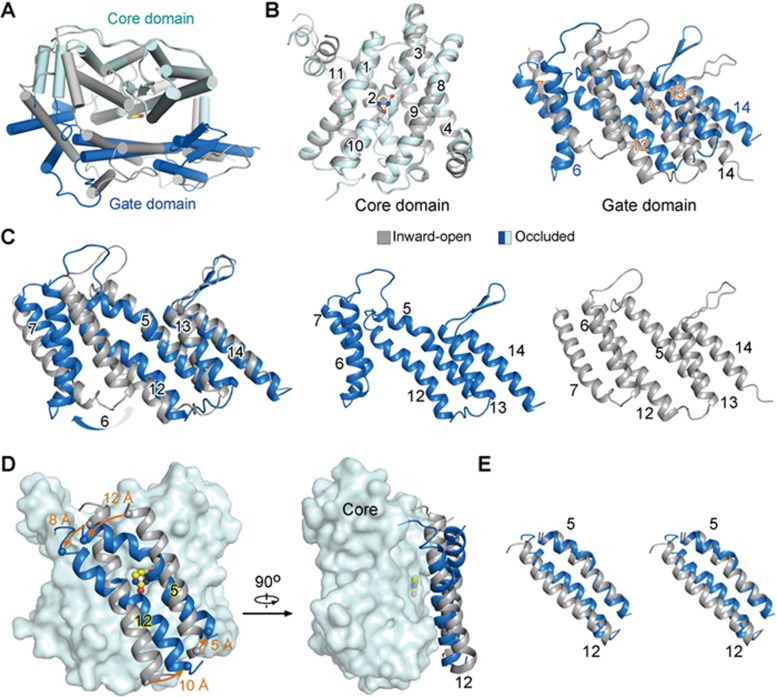
Conformational changes between UraA_IO_ and UraA_Occ_. **(A)** Structural comparison of UraA_IO_ and UraA_Occ_. The two structures are superimposed relative to the core domain. UraA_IO_ (PDB accession code: 3QE7) is colored grey and UraA_Occ_ is domain-colored. **(B)** The core domain and the gate domain undergo relative rotation around an axis that is roughly perpendicular to their interface. The core domain remains rigid in these two conformations, while the gate domain undergoes both inter- and intra-domain shifts when UraA_IO_ and UraA_Occ_ are superimposed relative to the core domain. **(C)** Intra-domain rearrangement of the gate domain. The two structures are superimposed relative to the gate domain in the left panel, and the individual gate domain structures of UraA_Occ_ and UraA_IO_ are shown in the middle and on the left, respectively. **(D)** Conformational shifts of TM5 and TM12 between UraA_IO_ and UraA_Occ_. The two structures are superimposed relative to the core domain. The translational distances of the Cα atoms of the terminal residues on TM5 and TM12 between the two structures are indicated. **(E)** Discordant conformational changes of TM5 and TM12. A stereoview of the superimposed TM5 and TM12 in the two structures is shown here.

Pronounced relative motions between the core domain and gate domain as well as intra-domain shifts of the gate domain are observed between the inward-open and occluded UraA structures (Figure 2A, 2B; Supplementary information, Movie S1). The core domains in the two structures can be superimposed with a root-mean-square deviation (RMSD) value of 0.38 Å over 202 Cα atoms, suggesting domain rigidity during the state transition (Figure 2B). In contrast, the gate domain undergoes considerable intra-domain rearrangements (Figure 2C).

Among the six TMs in the gate domain, TMs 6 and 7 show poor density in the previous UraA_IO_. In particular, TM6 has almost no contact with the rest of the protein structure. Comparison of the UraA_IO_ and the UapA structure in the inward-facing conformation shows good alignment except for TM6 and 7. We could not rule out the possibility that the local conformations of TMs 6 and 7 were potentially disrupted during protein extraction or crystallization. We therefore refrain from over-interpreting the conformational changes of these two segments, but focus on TMs 5 and 12 whose conformational changes directly determine the accessibility to the substrate-binding site.

The structural shifts of TMs 5 and 12 represent a combination of multiple modes of motions (Figure 2C and 2D; Supplementary information, Movie S1). When the two structures are compared relative to the core domain, both TMs 5 and 12 rotate around an axis that is nearly perpendicular to the interface between the core and gate domains. In addition, TMs 5 and 12 are straight α-helices in UraA_IO_, but both bent in UraA_Occ_ with a more prominent kink in TM5 (Figure 2E). Accompanying the bending and rotation, the periplasmic segment of TM5 moves towards, whereas the cytoplasmic segment of TM12 is displaced away from the core domain, resulting in the switch from occluded to inward-open state (Figure 2D; Supplementary information, Movie S1).

It is noted that the distinct chemical compositions of the gate domain and the core domain provide the molecular basis for their respective flexibility and rigidity (Supplementary information, Figure S2). The gate domain is literally one layer of transmembrane helices enriched of hydrophobic residues. In contrast, the core domain contains three layers of transmembrane segments that are bound tightly by extensive hydrogen bonds (H-bonds) directly between polar residues and mediated by extraordinary number of water molecules infiltrated throughout the core domain (Supplementary information, Figure S2B).

A similar feature was observed in the high-resolution structure of the human glucose transporter GLUT3, in which the C domain has a hydrophobic interior and the N domain contains a chain of water-mediated hydrogen bonds. Consistently, structural comparison between the outward-facing GLUT3 and inward-open GLUT1 reveals rigid-body rotation of the N domain and local rearrangement within the C domain^25,26^. So is XylE, the bacterial homologue of GLUTs^27,28,29^. However, it should be noted that the inter-domain motions are relative. The domain rigidity refers to the lack of intra-domain rearrangement.

### Uracil coordination and proton coupling

**Figure 3:**
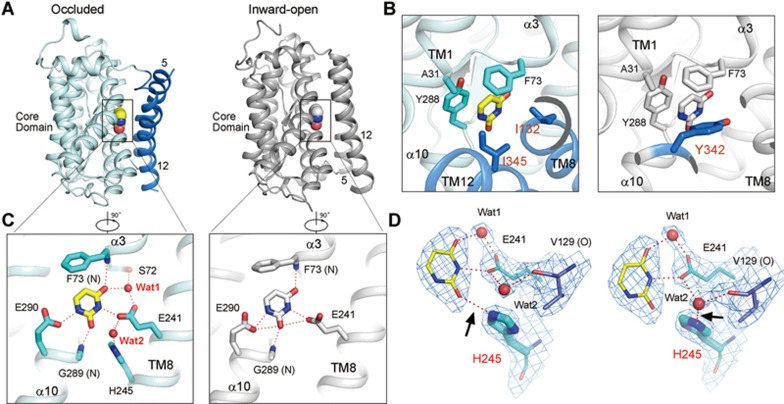
Substrate coordination. **(A)** Uracil is coordinated primarily by the core domain in both UraA_IO_ and UraA_Occ_. The bound ligand is shown as spheres in the two structures. For visual clarity, only TMs 5 and 12 are shown for the gate domain. **(B)** The hydrophobic residues that surround the bound uracil in UraA_IO_ and UraA_Occ_. Note that the gate domain residue Tyr342 in UraA_IO_ is replaced by Ile345 in UraA_Occ_ for substrate coordination. **(C)** Comparison of the coordination of uracil through polar interactions in UraA_IO_ and UraA_Occ_. Water molecules are shown as red spheres. Hydrogen bonds (H-bonds) are represented by red dashed lines. **(D)** Glu241 and His245 may play an important role in proton coupling. The distinct rotamer conformations of His245 may result in alternative H-bond formation with uracil (left) or Wat2 (right). His245 may play an important role in proton-coupling. The 2F_O_-F_C_ electron density map, shown as blue mesh, is contoured at 1σ.

In the previous UraA_IO_ structure, the bound uracil is primarily accommodated by the core domain. In UraA_Occ_, the substrate remains in the indentation on the surface of the core domain (Figure 3A; Supplementary information, Figure S1C). Due to the pronounced structural shifts of the gate domain, Tyr342 on TM12, the only residue from the gate domain that contributes to substrate coordination in UraA_IO_ is displaced by Ile345 in UraA_Occ_ (Figure 3B; Supplementary information, Movie S1). In contrast, the coordination of uracil by the core domain remains nearly unchanged in the UraA_IO_ and UraA_Occ_ structures owing to the rigidity of the core domain. The functionally essential residues, Glu241 and Glu290, contribute multiple H-bonds with uracil^16^. The backbone amide groups of Phe73 and Gly289 also directly interact with uracil (Figure 3C).

The improved resolution of UraA_Occ_ allows reliable assignment of water molecules, which reveals multiple water-mediated H-bonds for substrate coordination (Figure 3C). One water molecule (Wat1) bridges the indirect H-bonds between the side groups Ser72 and Glu241 with the O4 group of uracil. Another water molecule (Wat2) is in the vicinity of uracil and the functionally essential residues Glu241and His245^16^. Contiguous electron densities are observed between Wat1 and the carboxylate of Glu241 and between Wat2 and the carbonyl oxygen of Val129, which resides in the middle of TM5 in the gate domain (Figure 3D). Such contiguous densities may represent H-bonds. In the previously reported inward-open UraA structure, a detergent molecule β-nonyl-D-glucoside (β-NG) cuts into TMs 5 and 12 with its polar moiety involved in uracil coordination. Comparison of the two structures shows that the C3 hydroxyl of β-NG occupies the position of Wat2. Other than that, the conformations of the surrounding polar residues remain unchanged (Figure 3C; Supplementary information, Figure S3).

The presence of the two water molecules facilitates the establishment of an H-bond network involving the bound uracil, the functionally essential residues Glu241 and His245 in the core domain, and a backbone carbonyl oxygen in the gate domain (Figure 3C and 3D). Such an H-bond network may provide a path for H^+^ translocation. It may also represent the molecular basis for coupling the substrate transfer and proton translocation to the conformational changes of the transporter.

To investigate the role of Glu241 and His245 in H^+^ translocation, we performed molecular dynamic (MD) simulations starting from the crystal structure of dimeric UraA_Occ_ placed in a lipid bilayer (Supplementary information, Figures S4, S5, Movies S2, S3). To model different stages of the translocation, the proton was assigned to either Glu241 (termed system A) or His245 (termed system B) in both monomers in two independent MD simulations. The simulation of system A with protonated Glu241 reveals a local rearrangement that results in hydration of the substrate-binding site in both monomers, eventually leading to the disruption of the H-bond network that keeps uracil in its bound orientation (Supplementary information, Figures S4A and S4B, Movie S2). In contrast, in system B, protonation of His245 further stabilizes the H-bond network observed in the crystal structure, including direct uracil interactions of Glu241 and Glu290 (Supplementary information, Figures S5A and S5B, Movie S3). In our simulation, protonated His245 does not directly coordinate with uracil, but rather stabilizes the position of Glu241. His245 undergoes a reorientation (of ∼60° in χ_2_) relative to the crystal structure in both monomers and interacts with Glu241, which in turn forms a H-bond with the N3 group of uracil, and maintains the orientation of Wat1 to form an indirect H-bond with the O4 group of uracil (Supplementary information, Figures S5B, Movie S3). Interestingly, in one UraA protomer of this system, the original Wat1 from crystal structure is displaced by another water molecule that takes its place, revealing the dynamic nature of the H-bond network. These simulations shed light on the molecular basis of proton translocation mediated by Glu241 and His245, and highlight its role in substrate binding in UraA.

### Dimeric assembly of UraA_Occ_ in the structure

**Figure 4:**
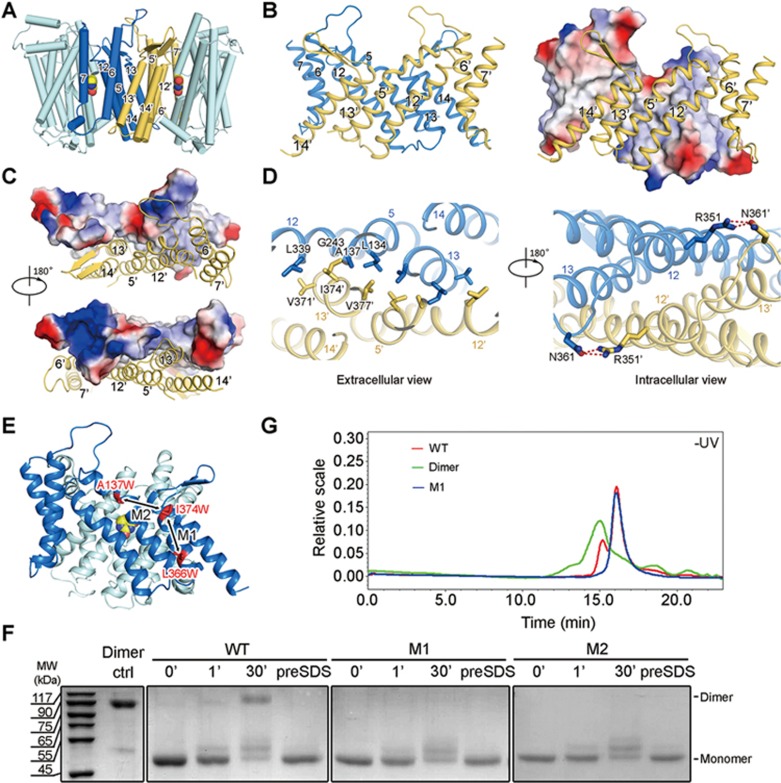
Dimeric assembly of UraA_Occ_ in the crystal and a monomer-dimer equilibrium of UraA in detergent micelles. **(A)** Two UraA protomers from adjacent asymmetric units form a homo-dimer mediated through their respective gate domains. The core domains in the two protomers are colored cyan, while the gate domains are colored blue and light orange, respectively. **(B)** The gate domains in the two protomers stabilize each other. The individual gate domain is a thin layer of transmembrane helices and has a relatively narrow cross section from intramembrane view. **(C)** The surface contours of the two gate domains are highly complementary to each other. The extracellular (top) and intracellular (bottom) views of the two gate domains in the dimeric UraA_Occ_ are shown here. The two gate domains are shown as surface electrostatic potential and ribbon cartoon, respectively. **(D)** The dimer interface of UraA_Occ_ is dominated by van der Waals interactions. The two UraA molecules interact with each other mainly through hydrophobic residues on TM 5/12/13 (left panel). There is only one pair of polar interactions between Arg351 and Asn361 between the two protomers (right panel). **(E)** Engineering of constitutive monomeric variants. Two pairs of double point mutations were generated based on the structural analysis, namely M1 (I374W & L366W) and M2 (A137W & I374W). **(F)** Crosslinking analysis of UraA variants using glutaraldehyde. The WT, M1, and M2 variants of UraA were subjected to 0.5% glutaraldehyde (w/v) with indicated reaction durations before quenching and subsequent SDS-PAGE. *Dimer*: an engineered constitutive dimer with two UraA molecules tandemly connected by a short peptide linker. “preSDS”: the protein was treated with 2% SDS (w/v) before addition of the crosslinker. **(G)** Static light scattering (SLS) analysis of UraA variants. The SLS characterizations of WT, M1, and the constitutive dimer are shown here.

Whereas UraA_IO_ appeared to be a monomer in the crystal structure, the UraA_Occ_ molecules from adjacent asymmetric units form a homodimer (Figure 4A; Supplementary information, Figure S6). The dimerization of UraA, which involves ∼2 400 Å^2^ buried surface area, is mediated through the gate domains (Figure 4B; Supplementary information, Figure S6). For clarity, we label the structural elements in the second protomer with an apostrophe ('). The dimeric interface is highly complementary, involving extensive hydrophobic residues on TMs 5/12/13 in each protomer. TMs 5 and 12 from one protomer contact TM13' in the other through van der Waals contacts (Figure 4C and 4D). The only hydrogenbonds are observed between the side groups of Arg351 on TM12 in one protomer and Asn361', which is located on the connecting loop between TMs 12' and 13', in the other protomer (Figure 4D).

The extensive interface indicates that the dimeric assembly may not be due to crystallographic symmetry. To elucidate the physiological oligomerization state of UraA, we carried out systematic *in vitro* and *in vivo* characterizations.

### UraA exists in equilibrium between monomer and dimer in detergent micelles

When purified through size exclusion chromatography (SEC) in a variety of detergents such as β-dodecyl-D-maltoside (DDM), LDAO, and NG, UraA always elutes in two peaks, suggesting the presence of more than one oligomerization state. Considering the structural observation, we speculated that purified UraA might exist in equilibrium between monomer and dimer. To investigate this, we generated two types of control proteins, namely, two constitutive monomers and one constitutive dimer of UraA.

To obtain constitutive monomers, a couple of interface residues were replaced by the bulky residue Trp for each mutant (M1: L366W & I374W; M2: A137W & I374W), a strategy that was used to generate the monomeric Cl^−^:H^+^ exchanger CLC^30^ (Figure 4E). To obtain a constitutive dimer, two UraA molecules were connected in tandem with a short peptide linker because both the N and C termini of UraA are on the cytoplasmic side of the membrane. Various lengths of the linkers, ranging from 2 to 12 amino acid residues (aa), resulted in proteins that behaved similarly. We will focus on the construct with a 12-aa linker for illustration hereafter (Figure 4F, “Dimer”).

The oligomerization states of the wild type (WT), M1, M2, and Dimer proteins were examined by crosslinking and static light scattering (SLS) approaches. When treated with 0.5% (w/v) glutaraldehyde^30,31^, approximately two-thirds of WT UraA protein migrated to a position corresponding to ∼90 kDa on sodium dodecyl-sulfate (SDS)-PAGE after 30-min treatment, suggesting the formation of crosslinked dimer. In contrast, both M1 and M2 failed to be crosslinked (Figure 4F). The crosslinking experiment supports dimer formation of WT UraA in detergent micelles. When applied to SLS, WT UraA eluted in two peaks, suggesting the existence of at least two oligomeric species. Whereas the monomeric variants were eluted as a single peak corresponding to the later peak of WT UraA, the UraA-Dimer was predominantly eluted at fractions slightly earlier than the first peak of WT UraA (Figure 4G).

The crosslinking and SLS examinations suggest the presence of both monomeric and dimeric UraA in detergent micelles. Different detergents, such as β-nonyl-D-glucopyranoside (NG), β-decyl-D-maltoside (DM), DDM, and Fos-choline 10, were tested and the conclusion remained the same.

Notably, among the transporters that share similar folds with UraA, UapA is a dimer with an extensive interface of up to 6 000 Å^2^ in the crystal structure, and dimerization is required for its cellular function^21,22^. Band 3 and AtBor1 also appear to be dimers^18,20^. SLC26Dg seems to stay as monomer in detergent micelles, although it can be crosslinked to a dimer^19^. We then sought to examine the functional relevance of dimerization for UraA.

### Dimer formation is required for the transport activity of UraA

**Figure 5:**
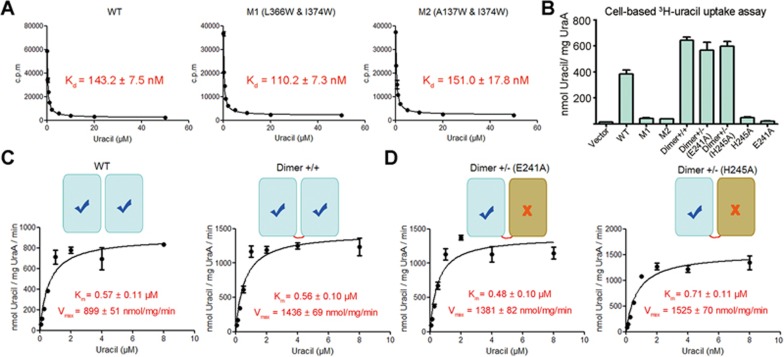
Dimer formation is required for the transport activity of UraA. **(A)** The UraA monomeric mutants retain similar binding affinities for uracil as WT. The binding affinities of UraA variants with uracil were measured using scintillation proximity assay (SPA). **(B)** Dimer formation of UraA is required for uracil transport. A cell-based ^3^H-uracil-uptake assay was performed following reported protocol^16^. “Dimer +/−” refers to the constitutive dimer with one WT molecule covalently linked to the variant containing the indicated single point mutation. **(C)** The transport activity of UraA variants. UraA-D exhibits enhanced *V*_max_, but similar *K*_m_ compared to WT UraA. **(D)** The dimer variants contain a loss-of-function mutation show similar transport activities to UraA-Dimer. Please refer to Methods for experimental details. All experiments were repeated at least three times. Error bars stand for SD.

It is noteworthy that the monomeric mutants of UraA bind to uracil with similar affinities as WT protein as measured in the scintillation proximity assay (SPA), confirming the correct folding of the monomeric mutants (Figure 5A). However, the transport activities of M1 and M2 were nearly abolished when examined in the cell-based uracil uptake assay^16^, suggesting that dimer formation is required for the transport activity of UraA (Figure 5B).

Based on the two observations that dimer is the functional form of UraA and WT UraA may exist in equilibrium between dimer and monomer, we reasoned that a constitutive dimer UraA would have higher transport activity than WT. Indeed, the UraA-Dimer exhibited an augmented activity, ∼70% higher than that of WT protein (Figure 5B). Note that the expression level of the UraA-Dimer is lower than WT or monomeric variants (Supplementary information, Figure S7). The transport activities were normalized against their expression levels. UraA-Dimer exhibited an enhanced *V*_max_, but similar *K*_m_ compared to the WT UraA (Figure 5C).

We then examined whether both protomers have to be functional for the transport activity. For this, we generated two additional constitutive dimer variants, each containing a loss-of-function point mutation, E241A or H245A^16^, in the second protomer. The expression levels of these two variants are similar to that of the UraA-Dimer (Supplementary information, Figure S7). Intriguingly, both variants showed similar *K*_m_ and *V*_max_ to that of the UraA-Dimer (Figure 5B-5D).

The functional characterizations of WT, monomeric, and dimeric UraA variants suggest that dimerization is required for the transport activity of UraA, although one functional protomer is sufficient for substrate translocation. However, whether the two protomers transport substrate independently of each other or in cooperation remains to be investigated.

### Structural comparison between Dimeric UraA_Occ_ and UapA

The structure of UraA_IO_ was determined as a monomer, precluding structural interpretation of the functional importance of dimerization. UraA and UapA share 23% and 40% sequence identity and similarity, respectively^16^. We thereby compared the structures of occluded UraA dimer and the inward-facing UapA dimer for clues.

**Figure 6:**
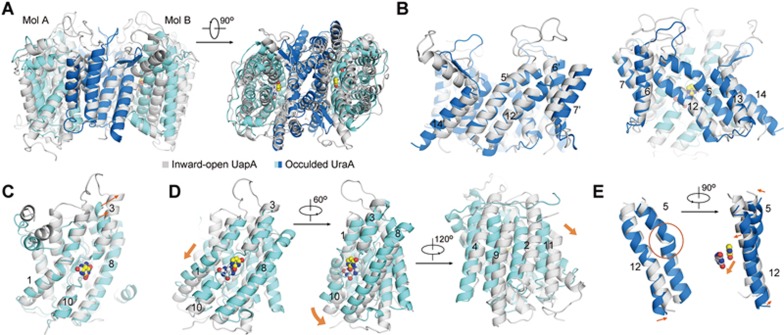
Structural comparison of the UraA_Occ_ dimer and the inward-facing UapA dimer reveals complex conformational changes for alternating access. **(A)** The overall structural comparison of UraA_Occ_ and UapA dimer. The structure of UapA (PDB code: 5I6C) is superimposed to that of UraA_Occ_ relative to the dimeric gate domain. **(B)** The gate domains of UraA_Occ_ and UapA can be largely superimposed. For clarity, the structural elements in the second protomer are labeled with an apostrophe. Left panel: structural superimposition of the dimeric gate domains in UraA_Occ_ and UapA. Right panel: only one protomer is shown. **(C)** The core domains of UraA_Occ_ and UapA are highly similar, with only minor local shifts. The core domains in one protomer of UraA_Occ_ and UapA are superimposed individually. **(D)** Pronounced shifts of the core domain between UraA_Occ_ and UapA when the two structures are compared relative to the dimeric gate domain. Three views are shown as indicated. The orange arrows indicate the shift orientations from UraA_Occ_ to UapA in each view. **(E)** Local conformational shifts of TM5 and TM12 between UraA_Occ_ and UapA. The substrates are shown as spheres in the right panel to indicate the relative positions of the core domains. The UraA is domain colored and UapA is colored silver. Please refer to Supplementary information, Movie S4 for the morph that illustrates the conformational changes between UraA_Occ_ and UapA.

Despite the distinct secondary structures of the extracellular paddle of TM13 and TM14 between UraA and UapA, their dimeric gate domains can be superimposed with the RMSD value of 3.9 Å over 182 Cα atoms (Figure 6A and 6B). Consistent with the sequence homology, the individual core domains of UraA_Occ_ and UapA can be overlaid with the RMSD value of 1.2 Å over 157 Cα atoms with the bound substrate completely overlapped (Figure 6C). However, when compared against the dimeric gate domains, the core domains between UraA_Occ_ and UapA undergo sophisticated domain-wise shift, resulting in the displacement of the substrate by ∼5 Å (Figure 6D; Supplementary information, Movie S4). When viewed from the gate domain side, the core domain in the same protomer undergoes a downward and leftward sliding from the occluded UraA to the inward-facing UapA, moving the bound substrate towards the intracellular side (Figure 6D, left panel). Meanwhile, the core domain and gate domain undergo a slight rotation around an axis that is perpendicular to their interface, similar to the shifts between UraA_Occ_ and UraA_IO_. The core domain also rotates around an axis that is approximately parallel to the interface between pore domain and gate domain, contributing to the downward shift of the substrate, which is translocated further away from the gate domain in UapA (Figure 6E; Supplementary information, Movie S4).

The motions of the core domains relative to the central dimeric gate domains appear to represent an elevator transport mechanism, as suggested from the comparison between Band 3 and AtBor1^32^. However, unlike the largely static scaffold domain in a typical elevator model, the gate domains display pronounced local rearrangements between UraA_Occ_ and UapA. Such differences may be in part attributed to the sequence variations between the two proteins. Nevertheless, the conformational changes of TM5 and TM12 between UraA_Occ_ and UapA are similar to those between the occluded and inward-open UraA structures, suggesting potentially conserved transport mechanism among the SLC23 members (Figures 2, 6E; Supplementary information, Movies S1 and S4).

## Discussion

**Figure 7:**
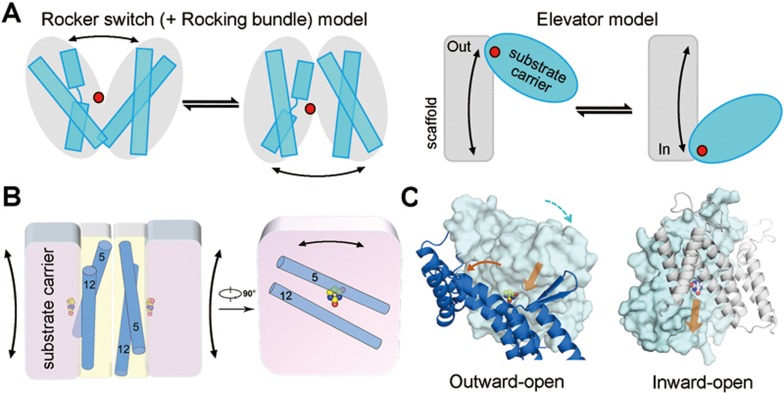
A putative working model for UraA and SLC23 members. **(A)** The prevailing models for the alternating access mechanism. The rocker switch model and rocking bundle model are combined on the left panel, and the elevator model is shown on the right. The red sphere indicates the substrate(s). Note that in both models, the approximate rotation axis of the two functional entities is approximately parallel to their interface (perpendicular to the page). **(B)** A putative working model of UraA. Dimerization may be required to achieve functional conformations of the gate domain, hence necessary for transport activity. The alternating access of each UraA protomer is accomplished by a combination of multi-mode motions of both the core domain and gate domain. **(C)** Putative paths for substrate entry and exit are indicated by semi-transparent arrows. The orange and cyan arrows in the left panel indicate the relative motions of the gate domain and the core domain that result in the exposure of the substrate-binding site to the extracellular side of the membrane.

The generic alternating access mechanism predicts that transporters undergo cycles of conformational changes to alternately expose the substrate-binding site(s) to the opposite sides of the membrane. Structural, biochemical, biophysical, and computational investigations in recent years suggested different modes of actions to realize alternating access mechanisms^33,34^: the “rocker switch” model exemplified by some Major Facilitator Superfamily (MFS) transporters and SWEET transporters^35,36^, the “rocking bundle” model exemplified by LeuT fold transporters^37,38,39,40^, and the “elevator” model exemplified by GlpT_Ph_^41,42^. In fact, many transporters, such as XylE and GLUTs, exhibit both rocker switch and rocking bundle motions for alternating access^25,26,27,28^. In all these models, completion of the transport cycle involves the conformational changes of one or both domains around an axis that is approximately parallel to the interface of two functional domains (Figure 7A).

Comparison of the structures of UraA in the occluded and inward-open state and with the inward-facing UapA dimer reveals a combination of multiple motions including both the elevator-like shift of the core domains and rocking bundle bending of the gate domains to achieve alternating access (Figures 2, 6, 7B). Even for the elevator-like motions, the substrate carrier (core domain) displays a more sophisticated motion trajectory than a simple rotation. The core domain undergoes both translational shifts and rotations that can be dissected to be around two orthogonal axes, one perpendicular and one parallel to the interface between the carrier and the scaffold (Supplementary information, Movie S4).

A unique structure feature for both UraA and UapA is the narrow cross section of the gate domain cutting through the membrane (Figures 4B and 7C). Relative rotations of a fairly small degree between the core domain and the thin gate domain, particularly the gating segments TMs 5 and 12, would result in the exposure of the central-binding site on the core domain to either side of the membrane (Figure 7C; Supplementary information, Movie S4). The planar shape of substrates such as uracil, vitamin C, and xanthine, would allow the substrate to conveniently slip in and out through a narrow cleft (Figure 7C). The gate domain is merely a thin layer of irregularly shaped TM segments, which may provide the flexibility for local conformational rearrangements during transport cycle. Dimerization may be required to stabilize essential functional conformations of the gate domains to facilitate the elevator-like movement of the core domains^21,43^ (Figures 4B and 7B). This model provides a structural interpretation for the necessity of dimerization as well as the independence of the functionality of the other protomer for the transport activity (Figure 5).

In sum, the biochemical, computational, and structural characterizations of UraA and other transporters reported here and previously provide the framework to decipher the transport mechanism of transporters in the SLC4, SLC23, and SLC26 families (Supplementary information, Figure S8). The high-resolution structure of UraA_Occ_ and MD simulations also identify Glu241 and His245 to be the potential candidates for proton-coupling. However, our present studies have not elucidated the mechanism for the proton-driven conformational changes of the transporter, which awaits further biochemical, biophysical, and computational examinations. It is also noteworthy that structural comparisons of different proteins should be interpreted with caution. Elucidation of the proton translocation-coupled structural shifts of the transporter and subsequent substrate association and dissociation requires structural determination of the same or closely related transporters in multiple transport states.

## Materials and Methods

### Protein preparation

The cDNA of UraA was amplified from *E. coli* strain O157:H7 and was subcloned into pET21b vector (Novagen). The UraA variants were generated by two-step PCR, overexpressed and purified as WT protein. The transformed *E. coli* BL21 (DE3) cells were grown at 37 °C to a cell density of 1.5 at A600 nm and induced with 0.2 mM isopropyl-β-D-thiogalactopyranoside (IPTG). After 16 h induction at 22 °C, the cells were collected, resuspended in the buffer containing 25 mM Tris-HCl, pH 8.0, and 150 mM NaCl, and disrupted using a French press with two passes at 10 000–15 000 p.s.i. The resulting supernatant after a centrifugation at 27 000× *g* for 10 min was further centrifuged at 150 000× *g* for 1 h to collect membrane fractions. The pellet of ultracentrifugation was resuspended and incubated with 1.5% (w/v) n-dodecyl-β-D-maltopyranoside (DDM, Anatrace) for 1 h at 4 °C. The lysate was centrifuged again at 27 000× *g* for 30 min and the supernatant was loaded to Ni^2+^-NTA affinity column (Qiagen). The resin was rinsed with the buffer containing 25 mM Tris-HCl, pH 8.0, 150 mM NaCl, 30 mM imidazole, 1 mM Uracil, and 0.2% n-decyl-β-D-maltopyranoside (DM, Anatrace) for three times. Then the target protein was eluted with wash buffer plus 250 mM imidazole, and concentrated to ∼10 mg/ml by centricon (Milipore) before being loaded to SEC (Superdex-200 10/30, GE Healthcare) in the buffer containing 25 mM Tris-HCl, pH 8.0, 150 mM NaCl, 1 mM Uracil and the indicated detergents. The peak fractions were collected for crystallization trials or biochemical characterizations.

### Crystallization

Crystals were grown at 18 °C using the hanging-drop vapor-diffusion method. Full-length UraA protein purified in the presence of 1.2% n-nonyl phosphocholine 9 (Fos-Choline 9, Anatrace) and 0.06% n-undecyl phosphocholine (Fos-Choline 11, Anatrace) gave rise to crystals in the space group C222_1_. Crystals of rod shape appeared after 3 days in the well buffer containing 24% PEG400, 100 mM MES-NaOH, pH 6.5, 100 mM NaF, 50 mM MgCl_2_, and 3 mM (NH_4_)_2_WS_4,_ and grew to well-diffracting crystal in more than 1 month. Crystals were directly flash frozen in a cold nitrogen stream at 100 K.

### Data collection and structure determination

The X-ray diffraction data were collected at SPring-8 beamline BL41XU with proposal No. 2012A1832 and processed with the HKL2000 package^45^. Further processing was carried out with programs from the CCP4 suite^46,47^. Data collection statistics are summarized in Supplementary information, Table S1.

Molecular replacement was performed with PHASER^48^ using a partial model of the *P*6_4_22 UraA structure^16^ as the initial search model. But the resulting phase solution was poor, preventing refinement. To solve this problem, a SAD dataset of the tungsten (W)-derived crystal was collected because (NH_4_)_2_WS_4_ was included in the crystallization solution. Using the previously obtained model as an input, the positions of the W atoms were determined by the PHASER SAD experimental phasing module. With identification of the W positions and the molecular-replacement model, better phases were generated using PHENIX AutoSol^49^. The automated model building was performed with ARP/wARP^50^ using the improved map. The model was built manually in COOT^51^ and the structure was refined with PHENIX^52^.

### Cell-based uracil-uptake assay

The *uraA*-deficient *E. coli* strain Keio Collection JW2482 *(F-, Δ(araD-araB)567, ΔlacZ4787(::rrnB-3), λ-, ΔuraA745:: kan, rph-1, Δ(rhaD-rhaB)568, hsdR514)* used in this assay was purchased from National BioResource Project (Japan). pQLINK vector^53^ was used for expression of WT and mutant UraA with 6-His tag at the C terminus. Western blot using an antibody against the His-tag was used to monitor the membrane expression levels of UraA variants. The amount of each UraA variant in the membrane fraction was estimated against a serial dilution of purified UraA with known concentrations on the same western blot^54^. The cell-based uptake assay was performed according to the reported protocol with some modifications^12^. The transformed *uraA*-deficient *E. coli* cells were grown at 37 °C to a cell density of *D*_600 nm_ 1.5 and induced with 50 mM IPTG at 37 °C for 30 min. Cells were collected, washed, and resuspended to an adjusted *D*_600 nm_ 2.0 in AB medium (please refer to http://openwetware.org/wiki/AB_medium for more details on the modified minimal medium). The harvested cells were incubated for 1 h at room temperature before the uptake assay.

To examine the uptake activities of the UraA variants, 2 μM [5,6-^3^H]-uracil (2 Ci mmol^−1^, American Radiolabelled Chemicals) was used in each assay. All the reactions were performed at room temperature. The reaction was performed for 30 s before an aliquot of cells was taken for rapid filtration through 0.45-μm cellulose acetate filter (Sartorius). The filter membranes were immediately washed with 2 ml ice-cold AB medium, dried, and taken for liquid scintillation counting. Cells transformed with pQLINK empty vector were used as negative control. The WT UraA and UraA variants were expressed and quantified following the same protocol.

The *K*_m_ and *V*_max_ of uracil uptake by WT and UraA variants were measured with the same protocol described previously^16^. Previous experiments showed that the accumulation of uracil was roughly linear within the first 30-60 s. Therefore, the initial velocities were measured at 30 s. All experiments were repeated by at least three times, and the data were fitted to the Michaelis-Menten equation, *V* = *V*_max_[UraA]/(*K*_m_+[UraA]), in GRAPHPAD PRISM 5.0 Demo.

### SPA-based binding assay

The SPA was performed exactly following the previously reported procedure^16^. All experiments were repeated at least three times and data are presented as mean ± SD. Nonspecific binding was subtracted from each data point. Data fitting was performed using GRAPHPAD PRISM 5.0 Demo.

### Crosslinking assay

Glutaraldehyde-mediated cross-linking of UraA was performed at room temperature. Samples (WT or UraA variants) at 1 mg/ml were treated with 0.5% glutaraldehyde, 150 mM NaCl and 50 mM HEPES, pH 7.5, for the indicated time course in 0.2% DM or, as a negative control, in 2% SDS. The reaction was quenched with 100 mM Tris-HCl. SDS-loading buffer was added, and the sample was analyzed by SDS-PAGE and visualized by Coomassie blue staining (16% gel).

### Construction of constitutive dimer

Constitutive dimer is composed of two UraA or UraA variant protomers connected by a linker with different lengths. The gene of one UraA protomer (named *uraA*1), plus an additional *Bam*HI cutting site (GGATCC), was built into pET21b vector with *Nde*I cutting site (CATATG) on 5′ and *Xho*I cutting site (CTCGAG) on 3′. The DNA sequence of the whole construct is pET21b: 5′-CATATG-*uraA*1-GGATCC-X_6_-CTCGAG-3′, X_6_ meaning six protective bases. We digested this new plasmid with *Bam*HI and *Xho*I to form a new vector. The DNA of another protomer (named *uraA*2) with a linker on its N-terminal was cloned into the new vector. The linker DNA containing a *Bam*HI cutting site (GGATCC) and several GlySer (GGCAGC) repeats. The sequence of whole constitutive dimer is pET21b: 5′-CATATG-*uraA*1-GGATCC-(GGCAGC)_n_-*uraA*2-CTCGAG-3′ (*n* = 0, 3, 5). Constitutive dimer in pQLINK is built in the same way as pET21b but with different restriction endonucleases. We used *Bam*HI, *Nde*I, *Not*I in pQLINK instead of *Nde*I, *Bam*HI, *Xho*I in pET21b.

### SEC-LS-UV SLS assay

The SEC-MAL system consists of a P900 HPLC pump (GE), a UV-2077 detector (Jasco) and a Tri Star Mini Dawn light scattering instrument (Wyatt). 100 μl of purified WT UraA or UraA variants proteins, each at 1 mg/ml, was injected into a WTC-030S5 gel filtration chromatography and eluted isocratically at 0.5 ml/min in a buffer containing 25 mM Tris pH 8.0, 150 mM NaCl, 1 mM Uracil, 0.2% DM. Data collection and analysis was performed with Astra 6 software (Wyatt).

### Modeling and simulation of UraA dimer in membrane

To elucidate the details of proton-coupling in substrate binding in UraA, we performed a series of MD simulations. The occluded state of UraA bound to uracil was used as the initial structure for the simulations. Missing residues (197-200) were added using MODELLER 9v10^55^. The UraA dimer was placed at the center of an equilibrated POPC bilayer (with X, Y lengths of 120 Å) generated with CHARMM-GUI^56^. Lipid molecules within 3.5 Å of the heavy atoms of the dimer were removed from the bilayer to avoid steric clashes with the protein, and the resulting protein-membrane structure was further inspected to check for any non-physical configurations (e.g., aromatic ring piercing by lipid tails).

After membrane embedding, four systems with different protonation states of key UraA residues involved in proton translocation (i.e., Glu241 and His245) were prepared. In two systems, both protomers (termed P1 and P2 hereafter) had protonated Glu241 (system A) or His245 (system B) in the uracil-binding site. For all systems, the orientation of His245 that directly interacts with uracil was selected initially. The resulting protein-membrane structures were solvated with the solvate plugin of VMD^57^, and the crystallographic waters already present in the UraA structure were preserved. The solvated systems were then neutralized with a 100 mM net concentration of Na^+^ and Cl^−^ ions with the autoionize plugin of VMD^57^. The resulting systems had dimensions of 120 Å × 120 Å × 120 Å and ∼161 000 atoms.

All systems were minimized for 2 000 steps, and equilibrated for 1 ns with the Cα of the dimer (except added residues 197-200) and the heavy atoms of uracil harmonically restrained (with force constant *k*=1 kcal/mol/Å^2^). Following this preparation step, all systems were simulated for 200 ns each without additional restraints.

### Protocol of MD simulation

All simulations were performed using NAMD2^58^. The CHARMM27 force field with cMAP^59,60^ corrections was used for the protein and the CHARMM36^61,62^ force field for nucleic acids and lipids was used for uracil and POPC, respectively. The TIP3P model was used for water^63^. All simulations were performed with the NPT ensemble with a time step of 2 fs. A constant pressure of 1 atm was maintained using the Nosé-Hoover Langevin piston method^64,65^. Temperature was maintained at 310 K using Langevin dynamics with a damping coefficient of 0.5/ps applied to all atoms. Nonbonded interactions were cut off at 12 Å, with smoothing applied at 10 Å. The particle mesh Ewald method^66^ was used for long-range electrostatic calculations with a grid density of > 1 Å^−3^.

### Accession codes

The atomic coordinates of UraA_Occ_ have been deposited in the Protein Data Bank under accession code 5XLS.

## Author Contributions

NY conceived this project. XY, GY, and NY designed all experiments. XY, GY, CY, KH, and HO performed the experiments. JB and ET performed the MD simulations. JJ, HF, and GL helped this project. XY, GY, and NY analyzed the data. XY, GY, JB, TW, ET, SL, and NY contributed to manuscript preparation. NY wrote the manuscript.

## Competing Financial Interests

The authors declare no competing financial interests.

## Supplementary Information

Supplementary information, Figure S1 The crystal structure of uracil-bound UraA in an occluded conformation.

Supplementary information, Figure S2 The distinct chemical compositions of the core domain and the gate domain.

Supplementary information, Figure S3 The detergent molecule β-NG replaces a water molecule, but appears to haveno effect on the local structures of polar residues.

Supplementary information, Figure S4 Simulation of dimeric UraA with protonated Glu241.

Supplementary information, Figure S5 Simulation of dimeric UraA with protonated His245.

Supplementary information, Figure S6 The surface contour of UraA dimer shown as electrostatic potential.

Supplementary information, Figure S7 The expression level of UraA variants.

Supplementary information, Figure S8 Structural comparison of UraA_Occ_ with AE1, SLC26Dg and Bor1.

Supplementary information, Movie S1 Conformational changes between UraA_IO_ and UraA_Occ_. The animation shows the relative rotation of the gate domain (blue) to the core domain (cyan). The TM5 and 12 in gate domain rotate around the axis perpendicular to the interface of the two domains, leading to the rearrangement of the residues around the uracil. Additionally, TM5 and 12 are bent in UraA_Occ_ compared to the UraA_IO_. Along with the bending and rotation, the TM5 periplasmic segment and the TM12 cytoplasmic segment moves in opposing direction toward the core domain, which results in the conformation changes between occluded and inward-open states. The morph is generated on the basis of the superimposition of the overall structures of UraA_IO_ and UraA_Occ_.

Supplementary information, Movie S2 Molecular Dynamics simulation of dimeric UraA with protonated Glu241. The animation shows the MD simulation of protonated Glu241 in the context of dimer. UraA dimer is placed in the POPC bilayer. Protonation of Glu241 reveals a local rearrangement leading to hydration of the substrate binding site in both protomers, which results in the disruption of H-bond network that keeps uracil in its bound orientation.

Supplementary information, Movie S3 Molecular Dynamics simulation of dimeric UraA with protonated His245. This animation shows the MD simulation of protonated His245 in the context of dimer. Protonated His245 plays a role in stabilizing the position of Glu241 and the H-bond network and forms an indirect H-bond with uracil through maintaining the orientation of Wat1. These observations highlight the important role of His245 in coordinating the uracil.

Supplementary information, Movie S4 Structural comparison between UraA_Occ_ and UapA. The animation shows the superimposition of UraA_Occ_ and UapA. In the context of dimer, both the core domains and the gate domains undergo complicated conformational changes. The core domains exhibit “up-and-down” movement and rotate relatively to the gate domain. At the same time, the gate domains rotate relatively to the core domains, which is exemplified by the rotation of TM5 and 12 in the gate domain. Overall, this animation shows the cooperation of the gate domains and the core domains during substrate translocation, which indicates the complexity of the transport mechanism. UraA_Occ_ is domain colored and UapA is colored silver. The morph is generated on the basis of the superimposition of the overall structures of UraA_Occ_ and UapA.

Supplementary information, Table S1 Statistics of data collection and refinement
