# Supplementary material for: Dimeric structure of the uracil:proton symporter UraA provides mechanistic insights into the SLC4/23/26 transporters
Source: Cell Res. 2017 Jun 16;27(8):1020–33. doi: 10.1038/cr.2017.83 (PMC5539350; doi:10.1038/cr.2017.83)
Supplement: Supplementary information, Figure S1 — The crystal structure of uracil-bound UraA in an occluded conformation. [file cr201783x1.pdf]

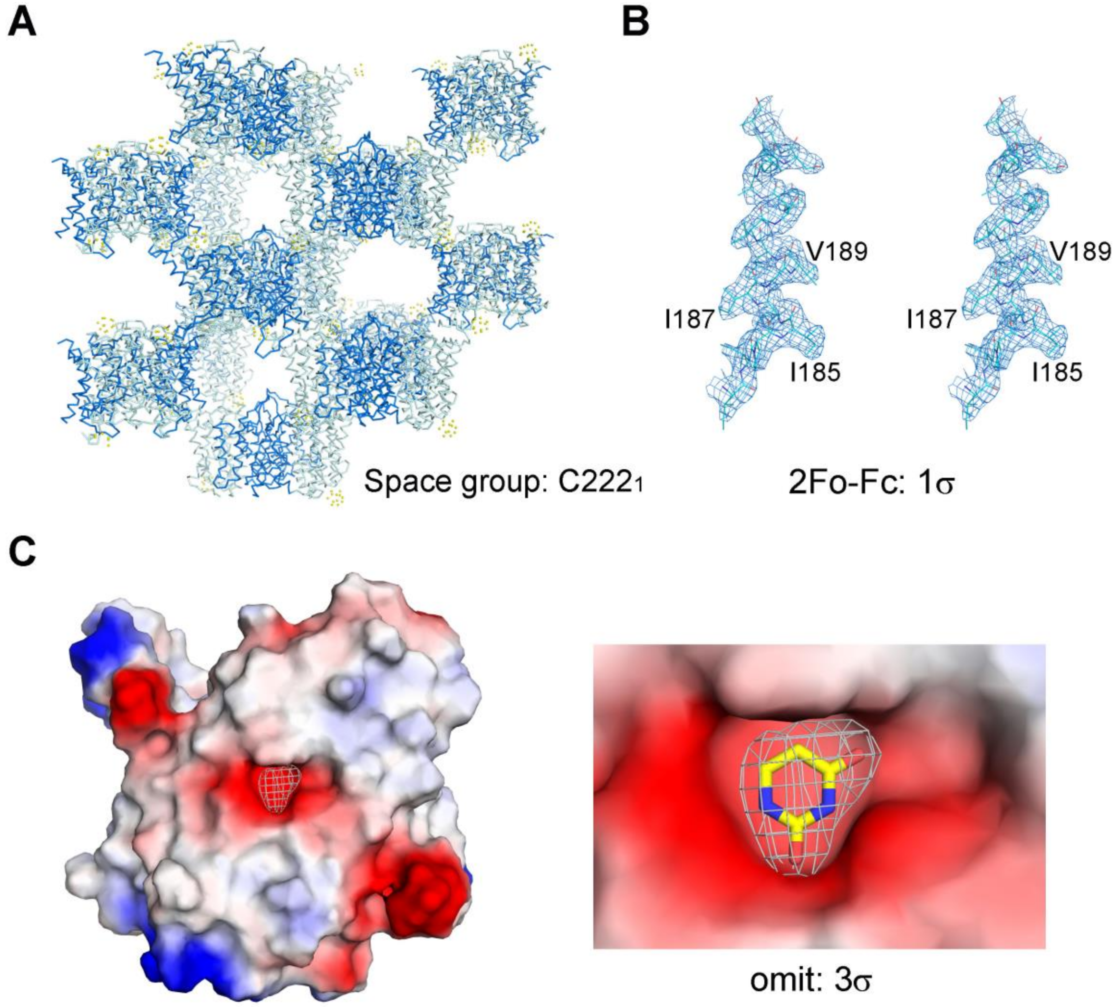

Supplementary information, Figure S1 The crystal structure of uracil-bound UraA in an occluded conformation.

(***A***) The lattice packing of UraA crystallized in the $C222_1$ space group. (***B***) The 2Fo-Fc electron density map for TM6 of UraA. The 2Fo-Fc electron density, shown in cyan mesh, is contoured at 1.0 σ and shown in stereoview. (***C***) The "omit" electron density map for uracil contoured at 3.0 σ. The surface electrostatic potential of the core domain is calculated in PyMoL.
