# Supplementary material for: Dimeric structure of the uracil:proton symporter UraA provides mechanistic insights into the SLC4/23/26 transporters
Source: Cell Res. 2017 Jun 16;27(8):1020–33. doi: 10.1038/cr.2017.83 (PMC5539350; doi:10.1038/cr.2017.83)
Supplement: Supplementary information, Figure S2 — The distinct chemical compositions of the core domain and the gate domain. [file cr201783x2.pdf]

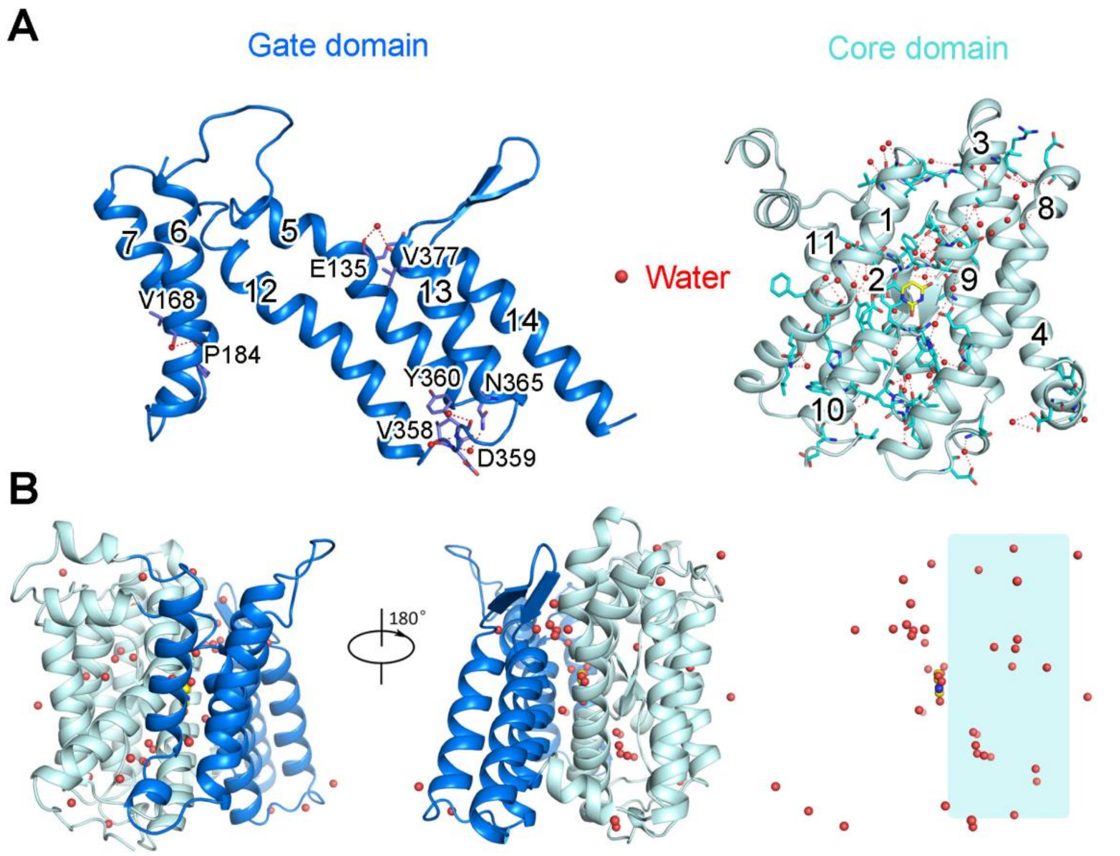

Supplementary information, Figure S2 The distinct chemical compositions of the core domain and the gate domain.

(***A***) The core domain is highly enriched of polar or charged residues whereas the gate domain is mainly composed of hydrophobic residues. Consequently, the core domain has numerous intra-domain hydrogen bonds between polar residues or mediated by water molecules. (***B***) The structure resolves an exceptionally large number of water molecules in the transmembrane region, mainly penetrating the core domain and on the interface between the core and gate domains. The water molecules are shown as spheres.
