# Supplementary material for: Dimeric structure of the uracil:proton symporter UraA provides mechanistic insights into the SLC4/23/26 transporters
Source: Cell Res. 2017 Jun 16;27(8):1020–33. doi: 10.1038/cr.2017.83 (PMC5539350; doi:10.1038/cr.2017.83)
Supplement: Supplementary information, Figure S3 — The detergent molecule β-NG replaces a water molecule, but appears to haveno effect on the local structures of polar residues. [file cr201783x3.pdf]

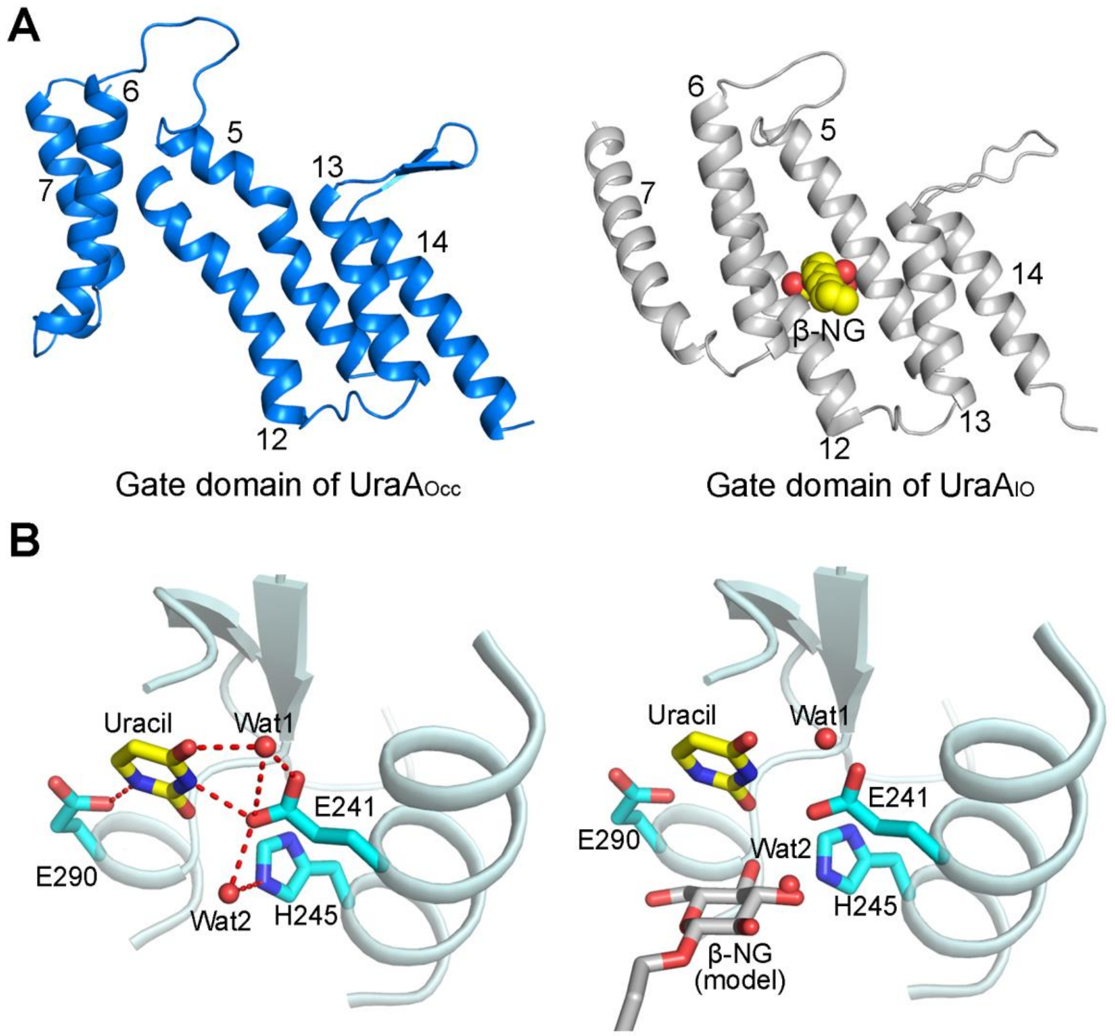

Supplementary information, Figure S3 The detergent molecule β-NG replaces a water molecule, but appears to have no effect on the local structures of polar residues. (***A***) The structure of the gate domain in the present occluded conformation and the previous inward-open conformation. The detergent molecule β-NG, which is sandwiched between TMs 5 & 12 in the inward-open structure, is shown as spheres. (***B***) One hydroxyl group of the β-NG molecule coincides with a water molecule for hydrogen-bonding to Glu241 and His245.
