# Supplementary material for: Dimeric structure of the uracil:proton symporter UraA provides mechanistic insights into the SLC4/23/26 transporters
Source: Cell Res. 2017 Jun 16;27(8):1020–33. doi: 10.1038/cr.2017.83 (PMC5539350; doi:10.1038/cr.2017.83)
Supplement: Supplementary information, Figure S4 — Simulation of dimeric UraA with protonated Glu241. [file cr201783x4.pdf]

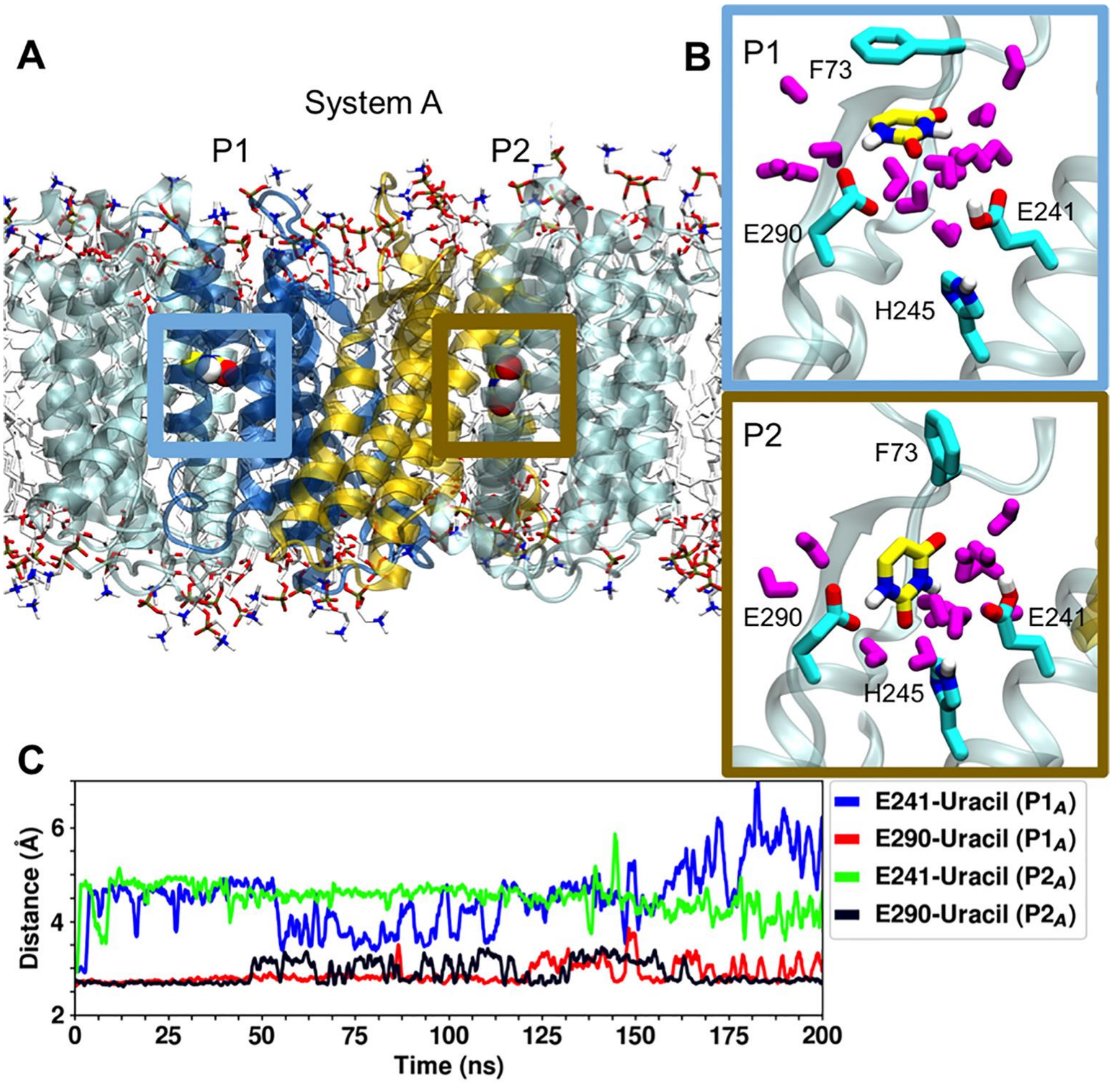

Supplementary information, Figure S4 Simulation of dimeric UraA with protonated Glu241.

(***A***) Representative snapshot (i.e., most often observed configuration) of UraA dimer in a POPC bilayer from simulation of system A. Lipids are shown in sticks. Protomers P1 and P2 are shown in cartoon, with core domains colored cyan, and the gate domains of P1 and P2 colored blue and light orange, respectively. Uracil molecule is shown as spheres. (***B***) Representative snapshot of the substrate binding site from simulation. Uracil and residues involved in uracil interactions and H^+-translocation are shown as sticks. Water molecules near uracil (within 5 Å), not including Wat1 and Wat2 are shown as magenta sticks. During the simulation, crystallographic waters Wat1 and Wat2 were displaced from their positions, and are thus not shown in the snapshots. (***C***) Time series of the distance between carboxyl group of Glu241 and Glu290, and N- groups of uracil in both P1 and P2 of system A.
