# Supplementary material for: Dimeric structure of the uracil:proton symporter UraA provides mechanistic insights into the SLC4/23/26 transporters
Source: Cell Res. 2017 Jun 16;27(8):1020–33. doi: 10.1038/cr.2017.83 (PMC5539350; doi:10.1038/cr.2017.83)
Supplement: Supplementary information, Figure S5 — Simulation of dimeric UraA with protonated His245. [file cr201783x5.pdf]

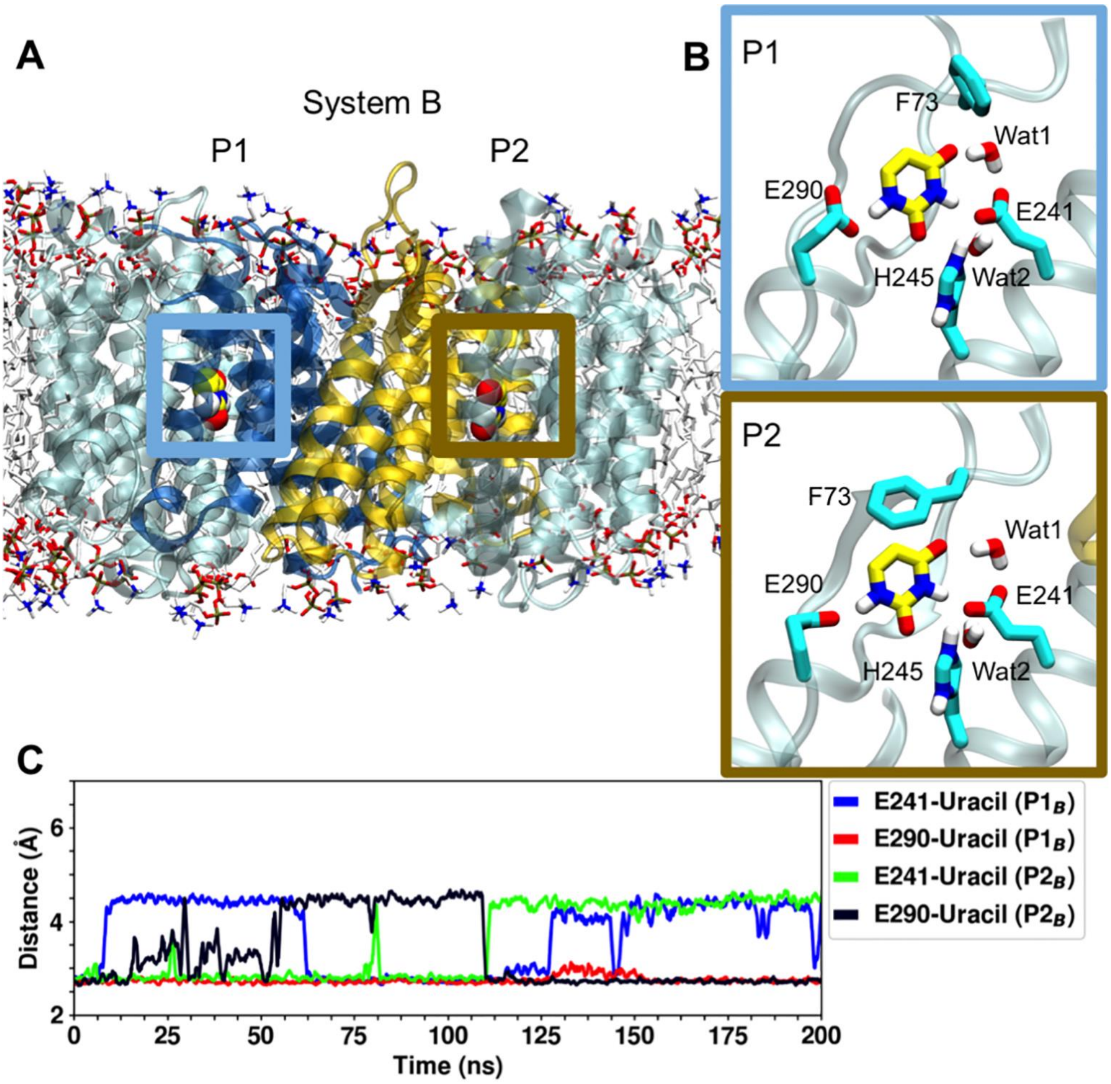

Supplementary information, Figure S5 Simulation of dimeric UraA with protonated His245.

(***A***) Representative snapshot of UraA dimer with protonated His245 in a POPC bilayer from simulation of system B. Lipids are shown in sticks. Protomers P1 and P2 are shown in cartoon, with core domains colored cyan, and the gate domains of P1 and P2 colored blue and light orange, respectively. Uracil molecule is shown as spheres. (***B***) Representative snapshots of the substrate binding site of protomers P1 and P2 in system B. Uracil and residues involved in uracil interactions and H^+-translocation are shown as sticks. Crystallographic waters Wat1 and Wat2 are shown in red/white stick representation. (***C***) Time series of the distance between carboxyl group of Glu241 and Glu290, and N- groups of uracil in protomers P1 and P2 of system B.
