# Supplementary material for: Dimeric structure of the uracil:proton symporter UraA provides mechanistic insights into the SLC4/23/26 transporters
Source: Cell Res. 2017 Jun 16;27(8):1020–33. doi: 10.1038/cr.2017.83 (PMC5539350; doi:10.1038/cr.2017.83)
Supplement: Supplementary information, Figure S6 — The surface contour of UraA dimer shown as electrostatic potential. [file cr201783x6.pdf]

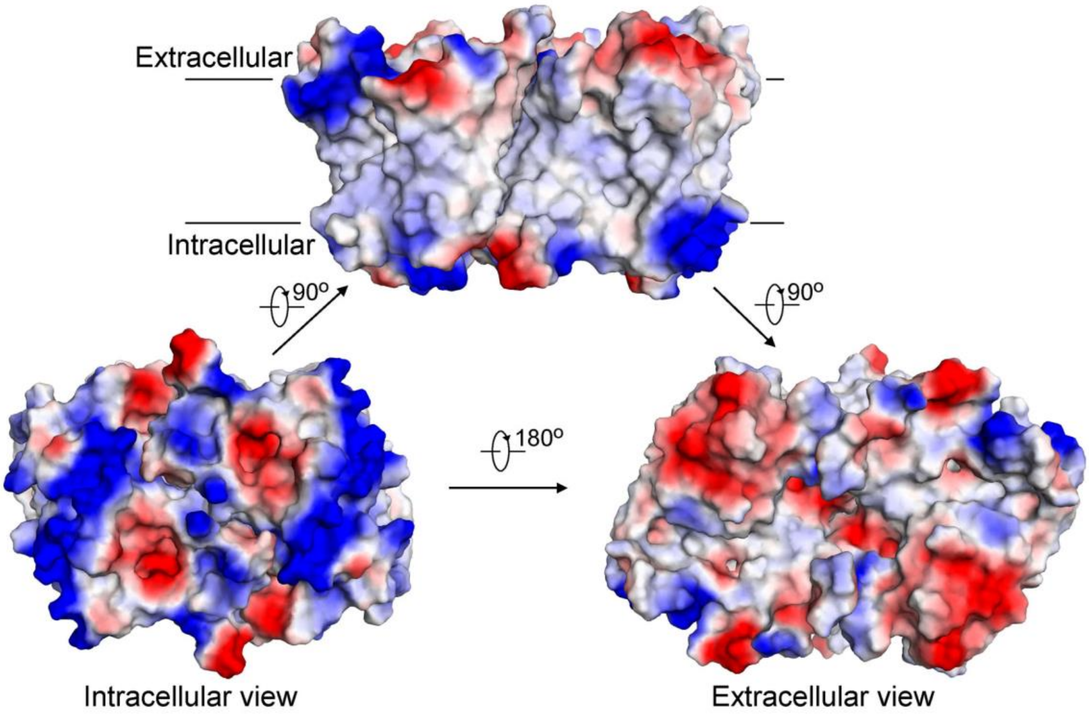

Supplementary information, Figure S6 The surface contour of UraA dimer shown as electrostatic potential.

The extracellular surface has a larger area than the intracellular side. The intracellular surface is enriched of positive charged residues, observing the “positive-inside” rule for integral membrane proteins.
