# Supplementary material for: Dimeric structure of the uracil:proton symporter UraA provides mechanistic insights into the SLC4/23/26 transporters
Source: Cell Res. 2017 Jun 16;27(8):1020–33. doi: 10.1038/cr.2017.83 (PMC5539350; doi:10.1038/cr.2017.83)
Supplement: Supplementary information, Figure S7 — The expression level of UraA variants. [file cr201783x7.pdf]

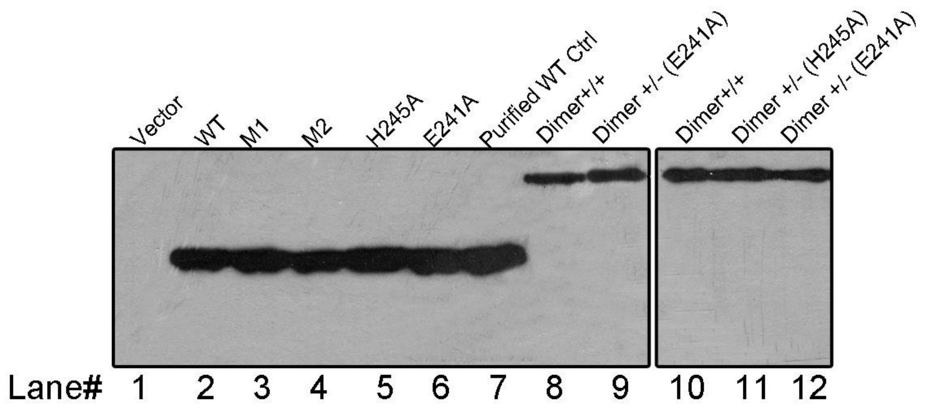

Supplementary information, Figure S7 The expression level of UraA variants. *E.coli* Δ*uraA* was transformed with the indicated UraA variants in the pQLINK vector. Same amount of cells were applied to SDS-PAGE followed by western-blot using antibodies against His_6 tag.
