# Supplementary material for: Dimeric structure of the uracil:proton symporter UraA provides mechanistic insights into the SLC4/23/26 transporters
Source: Cell Res. 2017 Jun 16;27(8):1020–33. doi: 10.1038/cr.2017.83 (PMC5539350; doi:10.1038/cr.2017.83)
Supplement: Supplementary information, Figure S8 — Structural comparison of UraAOcc with AE1, SLC26Dg and Bor1. [file cr201783x8.pdf]

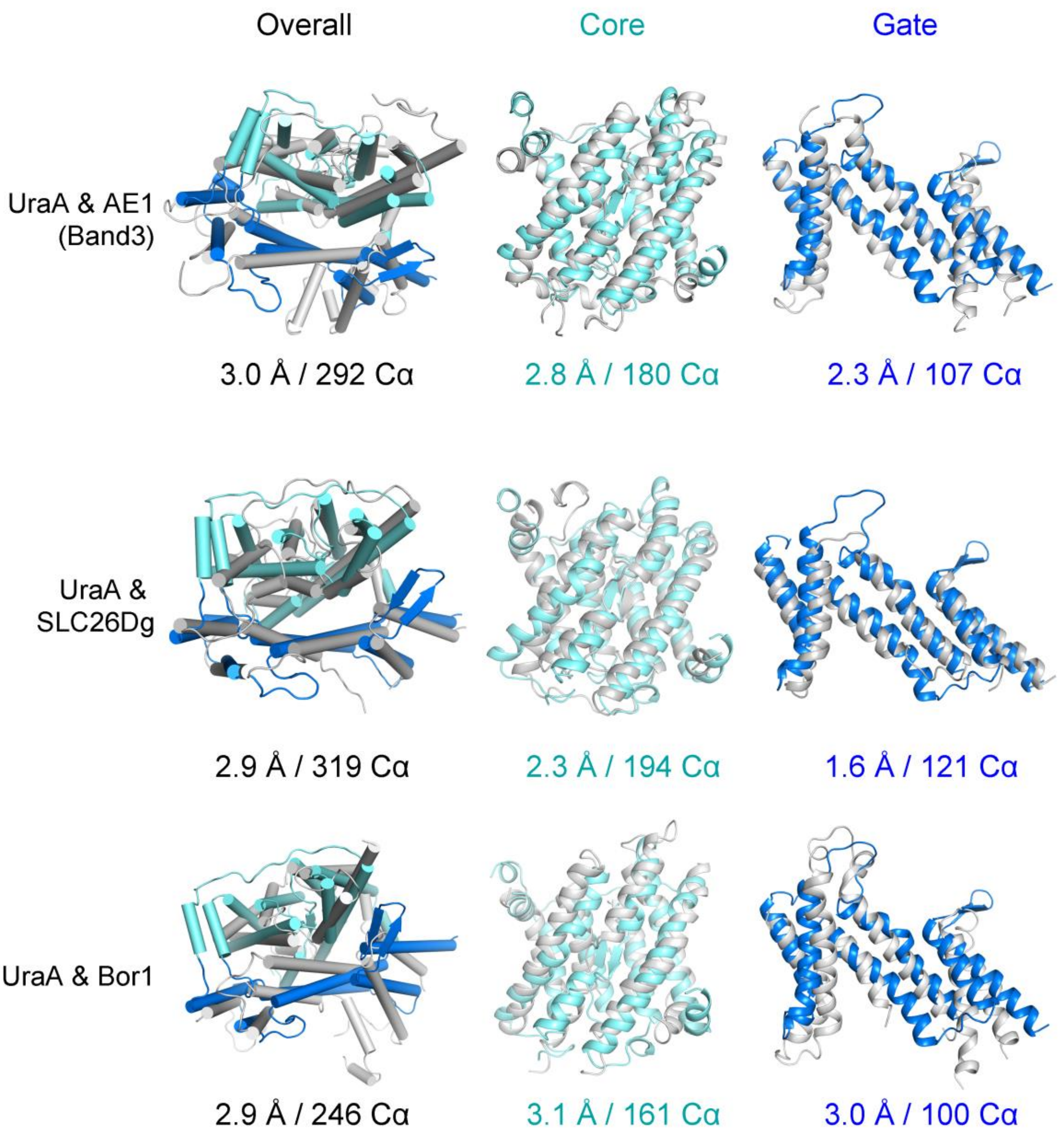

Supplementary information, Figure S8 Structural comparison of $UraA_{Occ}$ with AE1, SLC26Dg and Bor1.

The structures are compared relative to the overall structures (left), the core domains (middle), and the gate domains (right). UraA is domain colored and the compared proteins are colored silver. PDB codes: 4YZF for AE1, 5DA0 for SLC26Dg and 5L25 for Bor1.
