# Supplementary material for: Dimeric structure of the uracil:proton symporter UraA provides mechanistic insights into the SLC4/23/26 transporters
Source: Cell Res. 2017 Jun 16;27(8):1020–33. doi: 10.1038/cr.2017.83 (PMC5539350; doi:10.1038/cr.2017.83)
Supplement: Supplementary information, Table S1 — Statistics of data collection and refinement [file cr201783x13.pdf]

Supplementary information, Table S1 Statistics of data collection and refinement

**Data collection**	
Space Group	C 2 2 2_1
Unit Cell	
a, *b*, *c* (Å)	74.72, 134.73, 143.45
α, β, γ (°)	90.00, 90.00, 90.00
Number of molecules in ASU	1
Wavelength (Å)	1.0000
Resolution (Å)	40~2.50 (2.59~2.50)
R_{merge} (%)	11.7 (55.8)
I/σI	18.5 (2.8)
Completeness (%)	98.0 (98.5)
Number of measured reflections	81,965
Number of unique reflections	24,923
Redundancy	3.3 (3.3)
Wilson B factor (Å^2)	36.6
**Refine**	
R/R_{free} (%)	22.57/24.69
Number of atoms	
Protein main chain	1633
Protein side chain	1373
Protein all atoms	3006
Water molecules	42
Other entities	112
All atoms	3160
Average B value (Å^2)	
Protein main chain	49.8
Protein side chain	52.1
Protein all atoms	50.9
Water molecules	42.9
Other entities	65.7
All atoms	51.3
R.m.s deviations from ideal values	
Bonds (Å)	0.009
Angle (°)	1.451
Ramachandran plot statistics (%)	
Most favorable	93.3
Additionally allowed	6.7
Generously allowed	0
Disallowed	0

Values in parentheses are for the highest resolution shell. $R_{merge}=\Sigma_h\Sigma_i|I_{h,i}-I_h|/\Sigma_h\Sigma_i I_{h,i}$, where I_h is the mean intensity of the *i* observations of symmetry related reflections of *h*. $R=\Sigma|F_{obs}-F_{calc}|/\Sigma F_{obs}$, where F_{calc} is the calculated protein structure factor from the atomic model (R_{free} was calculated with 5% of the reflections selected).
